# Comparison of paperpoint and curette sampling of subgingival microbiome composition as analyzed by 16S rRNA gene amplicon sequencing

**DOI:** 10.1080/20002297.2017.1325260

**Published:** 2017-06-09

**Authors:** K. Beyer, B.W. Brandt, M.J. Buijs, J.G. Brun, W. Crielaard, A.I. Bolstad, E. Zaura

**Affiliations:** ^a^ Department of Clinical Dentistry, University of Bergen, Bergen, Norway; ^b^ Academic Centre for Dentistry Amsterdam (ACTA), Amsterdam, the Netherlands; ^c^ Department of Rheumatology, Haukeland University Hospital; ^d^ Department of Clinical Science, University of Bergen, Bergen, Norway

## Abstract

**Objective**: Our aim was to compare the subgingival microbiome composition in samples collected by curettes and paperpoints.

**Methods**: Subgingival plaque of rheumatoid arthritis patients with periodontitis (N=66) or gingivitis (N=15) was collected at two timepoints by sterile curettes and paperpoints. Unused sterile paperpoints were included as controls. The microbial DNA was processed for 16S rRNA gene amplicon sequencing. The data was processed and clustered into Operational Taxonomic Units (OTUs) and assessed by multivariate analyses.

**Results**: Unused paperpoints showed various levels of DNA contamination. The OTUs specific for unused paperpoints were classified as *Exiguobacterium, Enterococcus, Methylobacterium, Aquabacterium* and *Pseudomonas* and were removed from the dataset.

Microbial profiles of curette samples differed significantly from the paperpoint samples (PERMANOVA, p=0.0009, F=3.167). The paperpoint samples had significantly higher proportion of OTUs classified as *Streptococcus, Gemella, Parvimonas, Haemophilus, Aggregatibacter* and *Clostridiales* family XIII *incertae sedis* (p<0.05). Curette samples harbored higher proportion of *Corynebacterium, Prevotella, Selenomonas, Actinomyces* and *Treponema* (p<0.05).

Curette samples had significantly higher species richness (p=0.01) and Shannon Diversity Index (p=0.009) than the paperpoint samples.

**Conclusions**: Different subgingival plaque sampling techniques result in different microbiome profiles. Samples by paperpoints introduce microbial DNA contaminants and show underestimated microbial diversity compared to samples obtained by curettes.

